# Novel In Vitro Selection of *Trans*-Acting *BCL-2* mRNA-Cleaving Deoxyribozymes for Cancer Therapy

**DOI:** 10.3390/cells14130945

**Published:** 2025-06-20

**Authors:** Veera Vijaya Basamshetty, Vijay Kumar Gangipangi, Uppulapu Shravan Kumar, Santhosh Shanthi Bhupathi, Sridhar Reddy Kaulagari, Prashant Giri, Swapnil Sinha, Utpal Mohan, Konstantinos Sdrimas

**Affiliations:** 1 Department of Medical Oncology, West Virginia Cancer Institute, West Virginia University, Morgantown, WV 26505, USA; veeravijaya.basamshetty@hsc.wvu.edu; 2 Department of Pathology and Laboratory Medicine, Lewis Katz School of Medicine, Temple University, Philadelphia, PA 19140, USA; vijaya.kumar.gangipangi@temple.edu; 3 Department of Internal Medicine Carver College of Medicine University of Iowa, Iowa City, IA 52242, USA; shravankumar-uppulapu@uiowa.edu; 4 Department of Pharmaceutical Sciences, West Virginia University, Morgantown, WV 26505, USA; santhosh.shanthibhupathi@hsc.wvu.edu (S.S.B.); skaulagari@gmail.com (S.R.K.); 5 Department of Cell Stress Biology, Roswell Park Comprehensive Cancer Center, Buffalo, NY 14263, USA; prashant.giri@roswellpark.org; 6 IIT Guwahati TIDF BioNest, Guwahati 70005, India; ceo@bionestiitgtidf.com; 7 National Institute of Pharmaceutical Education and Research, Kolkata 70005, India

**Keywords:** DNAzymes, *BCL-2*, apoptosis, cancer, synthetic biology

## Abstract

The B Cell Lymphoma-2 (*BCL-2*) family proteins are central regulators of apoptosis, and their dysregulation is frequently associated with cancer progression and resistance to therapy. While small molecules like venetoclax have shown promise, nucleic acid-based therapeutics targeting *BCL-2* remain underexplored. Here, we report a novel in vitro evolution strategy to generate trans-acting RNA-cleaving DNAzymes targeting natural *BCL-2* mRNA without requiring covalent substrate-linking. Using a 50-base region of *BCL-2* mRNA as a selection target, we evolved several DNAzymes that demonstrate significant RNA cleavage activity. These DNAzymes downregulated *BCL-2* expression, induced apoptosis, and reduced cell viability in HepG2 and MCF-7 cancer cells. In vivo, our novel DNAzymes significantly suppressed tumor growth in a syngeneic mouse breast cancer model, with efficacy comparable to 5-Fluorouracil. This study presents a proof of concept for a novel strategy to evolve functional DNAzymes against native mRNA sequences and highlights their potential as gene-silencing tools in cancer therapy. Future studies will explore the therapeutic potential of these findings in cancer patients. Additionally, investigating the underlying molecular mechanisms in more complex cancer models will further validate the observed effects.

## 1. Introduction

Nucleic acids, beyond their role as carriers of genetic information, have emerged as versatile therapeutic agents capable of regulating gene expression. Among these, RNA-cleaving DNAzymes are synthetic single-stranded DNA molecules that catalyze site-specific RNA cleavage, offering advantages such as chemical stability, low production cost, and modular design. DNAzymes like 10–23 and 8–17, discovered through in vitro selection, have been widely studied for their ability to downregulate specific mRNA targets in disease contexts, including cancer, cardiovascular disorders, and viral infections [1,2,3,4].

The *BCL-2* protein family plays a pivotal role in regulating apoptosis, particularly by controlling mitochondrial membrane integrity and cytochrome c release [5]. Overexpression of anti-apoptotic members such as *BCL-2* is a hallmark of various cancers and is associated with treatment resistance [6,7,8]. Although small-molecule inhibitors like venetoclax [9,10] have been approved for *BCL-2* inhibition, alternative approaches such as nucleic acid-based gene silencing offer higher specificity and tunability.

In this study, we developed a novel in vitro selection approach for evolving RNA-cleaving DNAzymes that target natural, unmodified *BCL-2* mRNA. While DNAzymes such as 8–17 and 10–23 have been extensively studied using covalently linked RNA-DNA chimeras, our work introduces a novel, non-covalent in vitro selection methodology that allows for more physiologically relevant RNA-DNA interactions. This approach enables the selection of trans-acting DNAzymes that bind to natural mRNA substrates via Watson–Crick base pairing, mimicking endogenous hybridization dynamics. This design favors the selection of trans-acting DNAzymes that mimic physiological mRNA-DNA interactions. By avoiding artificial tethering, our strategy broadens the applicability of DNAzyme technology to native RNA targets and may enhance therapeutic translatability across diverse disease contexts, including cancer and other genetic disorders. We report two potent DNAzymes, DNZ-15 and DNZ-35a, which downregulate *BCL-2* expression in cancer cell lines and suppress tumor growth in a murine cancer model, thus providing a foundation for future DNAzyme-based cancer therapeutics.

## 2. Materials and Methods

### 2.1. In Vitro Selection of Trans-Acting mRNA Deoxy Ribozymes

A total of 50 bases of 5′-Biotin-tagged *BCL-2* mRNA (500 nM) (IDT, USA) was immobilized on 50 µL of M-280 streptavidin magnetic Dynabeads (Invitrogen). Then, 1 µM of ssDNA (IDT, USA) library was preheated at 95 °C for 5 min followed by flash chilling in ice and was added to the *BCL-2* mRNA immobilized on the streptavidin magnetic beads [11]. ssDNA library was incubated with immobilized mRNA for 20 min at room temperature, after which unbound library molecules were removed by wash buffer (10 mM Tris-HCl pH 8.0, 1 mM EDTA pH 8.0, and 2 M NaCl). The RNA cleavage reaction was carried out in a buffer containing 150 mM KCl and 2 mM MgCl_2_. The DNA-RNA hybrid was added to the reaction mixture, followed by incubation for 6 h [12]. The active DNAzyme pool was collected in the flow-through fraction, whereas the inactive molecules bound to the RNA were discarded along with the beads. The flow-through fraction containing the active pool of trans-acting mRNA-cleaving DNAzymes was further purified using a PCR purification kit (GenElute or equivalent). In some cases, an ethanol precipitation step was performed to concentrate and clean up the eluted DNA before proceeding to the next round of selection. The isolated DNAzyme pool was subjected to a PCR reaction with forward 5′-GGCTGGATGGGGCGTGT-3′ and 5′ Spacer-C18-tagged reverse primer. PCR was performed for 20 cycles (initial denaturation at 95 °C for 5 min and cycling at 94 °C, 30 s; 55 °C, 30 s; and 72 °C, 30 s). The amplified dsDNA DNAzyme pool was separated on denaturing 12% TBE Urea-PAGE [13]. The ssDNA band was excised from the gel and purified using the “crush and soak” method [14]. This selected DNAzyme pool was used for the next round of selection. The in vitro selection procedure was carried out for 10 iterative rounds. The incubation time for DNAzyme-mediated RNA cleavage was gradually reduced to 3 h in the last round. After a tenth round of in vitro selection, the selected DNAzyme pool was PCR-amplified, and the amplified dsDNA PCR fragments were cloned in a TA-Cloning vector (Pure Gene) [15]. The individual DNAzyme clones were sequenced at the 1st Base sequencing facility (Axil Scientific, Singapore).

### 2.2. Cell Culture and Transfection

The HepG-2 (human hepatocellular carcinoma) and MCF-7 (human breast carcinoma) cell lines were purchased from the National Centre for Cell Sciences (NCCS, Pune, India) and the mouse 4T1 (mammary carcinoma) cell line was purchased from ATCC (American Type Cell Culture, Manassas, VA, USA). HepG2 and MCF-7 were maintained in Dulbecco’s modified Eagle’s medium (DMEM, Gibco, Brooklyn, NY, USA) while the 4T1 cells were cultured in RPMI-1640 media at 37 °C with 5% CO_2_ and supplemented with 10% Fetal Bovine Serum (FBS) (Gibco), 1% Penicillin-Streptomycin (100 units/mL of Penicillin and 100 mg/mL of Streptomycin) (Gibco), and 0.25 μg/mL Amphotericin B (Gibco). The cells were harvested at ~60% confluency for transfection. All the transfection experiments were performed in reduced serum media (Opti-MEM^®^ I, Invitrogen, Carlsbad, CA, USA) with 200 nM of DNAzymes(s) using Oligofectamine Reagent (Invitrogen) according to the manufacturer’s protocol [16,17].

### 2.3. Quantitative Real-Time PCR

The cells were seeded at a density of 3 × 10^5^ cells/well in 6-well culture plates in a growth medium without antibiotics one day before the experiment. Transfection with DNAzymes was performed as mentioned above. The total RNA from the cells and tissues was isolated using TRIzol (Invitrogen) reagent [18,19,20]. For all the qPCR experiments, 1 μg of RNA was used to synthesize cDNA using a Verso cDNA synthesis kit (Thermo Scientific, Waltham, MA, USA). Real-time PCR was performed on a CFX Connect^™^ Real-Time PCR detection system (Bio-Rad, Hercules, CA, USA) in a 96-well plate using a program: 95 °C 5 min pre-denaturation and cycling at 94 °C, 10 s denaturation; 55 °C, 5 s annealing; and 72 °C, 10 s amplification. PCR was run for 40 cycles and all the samples were run in triplicates. *18s* RNA was used to normalize *BCL-2* expression and the relative fold expression values were calculated using the ∆∆C_t_ method [21]. The primers used for qPCR analysis are given in Table S1.

### 2.4. MTT Cytotoxicity Assay

For the cell viability assay, 1000 cells were seeded in a 96-well culture plate one day before the experiment. After DNAzyme transfection (200 nM in triplicates), 0.5 mg/mL of 3-(4, 5-dimethylthiazol-2-yl)-2, 5-diphenyltetrazolium bromide (MTT) solution (Sigma) was added at time points 12, 24, 36, 48, and 72 h. The cells were incubated for 3 h at 37 °C, after which the supernatant was removed and 200 μL of DMSO was added to dissolve the formazan crystals. After 10 min, the absorbance was recorded at 570 nm using a microplate reader (SpectraMax^®^, Molecular Devices, San Jose, CA, USA) [22,23].

### 2.5. Apoptotic Cell Imaging Using Confocal Microscopy

The HepG2 cells (4 × 10^4^) were seeded on poly-L-lysine-coated chamber slides one day before transfection. Then, 24 h after DNAzyme transfection (200 nM), the cells were washed with cold 1× PBS followed by the addition of 5 μL of FITC-Annexin V solution (Molecular Probes) and 100 μg/mL of Propidium Iodide solution (Molecular Probes) to each chamber. The cells were incubated for 15 min at room temperature, after which the cells were washed once with 1× Annexin binding buffer (Molecular Probes). The apoptotic/dead and live cells were imaged by a Leica Microsystems confocal microscope (TCS SP8) [24,25].

### 2.6. Animal Models and Tumor Implantation

Female BALB/C mice weighing approximately 15 to 20 gm were purchased from the NIN-Hyderabad with prior approval from the Animal Ethical Committee (Regno.1996/po/s/17/CPCSEA). The animals were quarantined and acclimatized in sheltered in vivo animal cages with proper temperature, light, feed, and water. Once the average weights of the animals reached 20 to 25 gms, the animals were divided into four groups (six animals per group). A 4T1 syngeneic breast cancer model was developed by injecting the pre-cultured 4T1 cells of the number 3 × 10^5^ cells in PBS per each animal. They were injected carefully in the 6th breast pad with 1 m of the insulin syringe [26,27]. The tumors were visible 10 days after the injection. The 3D tumor volumes were measured, and the average tumor volume of each group was recorded before and after the treatment of the DNAzymes (DNZ-15 and DNZ-35A Using High-Resolution Ultrasound and Color Doppler Imaging [28,29]. A total of 12 animals were finally included in the present study (n = 3 per group). The number of animals per group was determined based on the expected difference in tumor volume between the treated and control groups, ensuring sufficient statistical power to detect significant treatment effects.

The mice inclusion criteria were a uniform tumor volume ten days post-implantation. The animals with uneven tumor volumes were excluded from the analysis to ensure comparable starting conditions across the groups. No other exclusions were applied, which excluded the remaining 14 animals out of 36 animals from the study. Randomization was not formally used to allocate animals to the control and treatment groups. Allocation was performed manually, with efforts to ensure that the animals were distributed evenly across groups based on body weight and general health to reduce bias. All the animals were housed under the same environmental conditions (temperature, humidity, and light/dark cycle), and the treatments and measurements were carried out in a consistent manner. All the stages of the experiment—including group allocation, conduct of the experiment, outcome assessment, and data analysis—were performed by a single researcher. All the animal experiments were performed based on the Institution animal ethical committee guidelines.

### 2.7. In Vivo DNAzyme Studies

An experimental DNAzyme suspension was made in deionized water and injected at the tumor site at a concentration of 10 μg/animal (“naked DNA”). One group was injected with the 5-Flurouracil (5FU) at a concentration of 5 mg/kg body weight. Control animals were injected with sterile double-distilled water only. DNAzymes were administered on the 10th and 20th days after tumor induction. The animals were divided into four groups (n = 3: DNZ15, DNZ35a, 5-Fluorouracil, and control group) and the tumor volumes were measured on Day 0 (treatment initiated), Day 10, and Day 21 post-treatment. After 21 days, the tumors were carefully excised and washed with 1XPBS and dried on paper towels and the tumor weight and volumes were measured. The total study duration was 21 days. The positive control group received three injections of 5-fluorouracil (5FU) while the vehicle control group received three equivalent administrations **of** deionized water on the same schedule.

### 2.8. Immunohistochemistry

The tumor tissues were sliced at a diameter of 5 μm with histotome. The tissue sections were fixed in 10% formalin and embedded in paraffin wax. The sections were incubated with anti-*BCL-2* primary antibody (Merck) overnight at 4 °C followed by incubation with HRP-conjugated secondary antibody (Merck) for 2 h at room temperature. *BCL-2* expression was detected by adding a TMB substrate and the slides were imaged in the microscope (Leica Microsystems, Mannheim, Germany).

### 2.9. Western Blotting

The total protein was extracted from the tissue and was resuspended in RIPA buffer. A total of 40 μg/lane protein lysates was separated by 12% SDS-PAGE and transferred onto a nitrocellulose membrane (Bio-Rad) [9,30]. The membranes were blocked in 3% BSA for 2 h at room temperature and incubated at 4 °C overnight with anti-*BCL-2* (Cell Signaling) antibody at 1:1000 dilution. GAPDH (1:1000) (Cell Signaling) was used as an internal control. HRP-conjugated secondary antibody (1:10,000) (Cell Signaling) was added at room temperature for 2 h. ECL substrate (Clarity Max™, Bio-Rad) was added, and the bands were visualized by chemiluminescence (Fusion FX, Vilber Lourmat, France).

### 2.10. Statistical Analysis

All the graphs were constructed using GraphPad PRISM 8.0. Comparisons of the DNAzyme-transfected or -treated groups were performed with controls using the student *t*-test. The *p*-value of <0.05 was statistically significant.

## 3. Results

### 3.1. In Vitro Selection of BCL-2 mRNA-Cleaving DNAzymes

**Figure 1:**
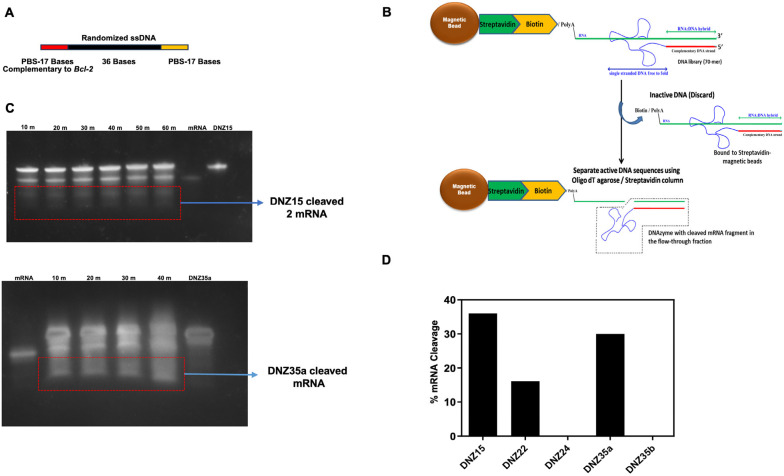
(**A**) Design of the ssDNA library. The forward and reverse primer binding sites (PBSs) are indicated in red and green, respectively, while the randomized 36-base sequence, which contains potential DNAzymes, is shown in black. (**B**) Schematic of the in vitro DNAzyme selection strategy. (**C**) The trans-cleavage of target *BCL-2* mRNA in vitro by DNZ15 and DNZ35a. (**D**) mRNA cleavage by the DNAzymes over time. The percentage of mRNA cleavage by the evolved DNZs (15, 22, 24, 35a, and 35b) was calculated using the densitometry intensity of the cleaved mRNA band.

To develop DNAzymes targeting *BCL-2* mRNA, we employed a novel trans-acting in vitro selection strategy designed to enrich catalytically active DNAzymes under near-physiological conditions. This approach utilized a 70-mer single-stranded DNA (ssDNA) library, containing a central 36-nucleotide randomized region flanked by 17-nucleotide primer binding sites (Figure 1A). The target substrate for selection was a 50-nucleotide biotinylated RNA fragment derived from the 5′ end of human *BCL-2* mRNA and identical to the mouse *BCL-2* mRNA, which was immobilized on streptavidin-coated magnetic beads to enable selective partitioning of active DNAzymes from the inactive pool (Figure 1B).

Unlike traditional cis-acting selection strategies, where the RNA and DNA are covalently linked, our trans-acting design allows for free interaction between the DNAzyme and RNA—more closely mimicking physiological interactions. Through ten iterative rounds of selection, washing, and amplification, we enriched a pool of ssDNA molecules with increasing catalytic activity.

After the final round, the DNAzyme pool was cloned and sequenced, resulting in the identification of five distinct DNAzyme candidates: DNZ-15, DNZ-22, DNZ-24, DNZ-35a, and DNZ-35b. These were synthesized and screened individually for trans-cleaving activity against the *BCL-2* RNA substrate under in vitro conditions using denaturing PAGE analysis. Time-course assays revealed that among the tested molecules, DNZ-15 and DNZ-35a demonstrated the most efficient catalytic activity, cleaving approximately 35% and 30% of the substrate RNA, respectively, within 80 min of incubation (Figure 1C,D).

### 3.2. DNZ-15 and DNZ-35a Downregulate BCL-2 Expression in Cancer Cell Lines

**Figure 2:**
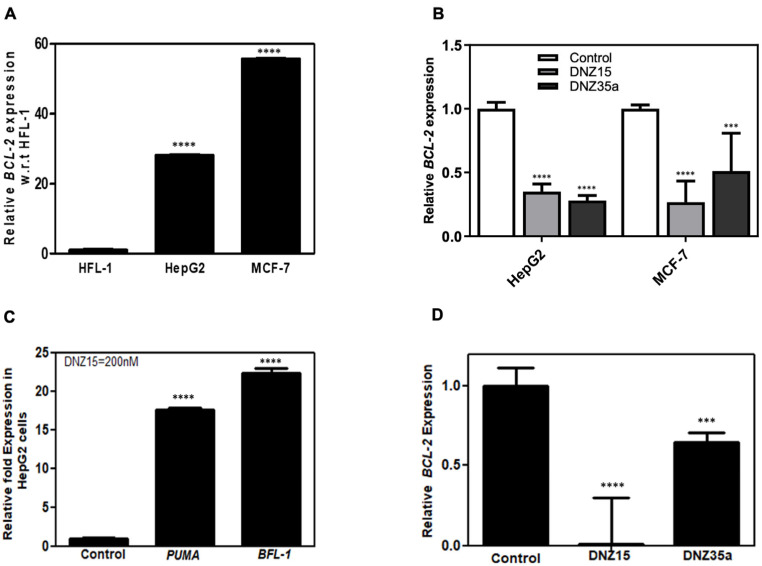
(**A**) Relative *BCL-2* mRNA expression in cell lines HFL-1 (human lung fibroblast), HepG2 (human hepatic carcinoma), and MCF-7 (human breast cancer). (**B**) DNAzyme-mediated *BCL-2* mRNA downregulation in HepG2 and MCF-7 cell lines in 24 hrs by DNAzymes 15 and 35a at a concentration of 200 nM. (**C**) Upregulation of *BCL-2* pathway proteins PUMA and BFL-1 in HepG2 cells after treatment with DNZ15 (200 nM). (**D**) *BCL-2* mRNA expression levels in cultured 4T1 cells transfected with 200 nM of DNZ15 and DNZ35a. mRNA levels in (**B**), (**C**), and (**D**) are expressed relative to the control (mock-transfected) set to 1. Error bars represent the mean ± SD from three separate experiments performed in triplicates. Significance levels are indicated as *** *p* < 0.001 significant and **** *p* < 0.0001 highly significant.

To assess the functional activity of our evolved DNAzymes in a cellular context, we evaluated the expression of *BCL-2* mRNA in multiple cancer cell lines following DNAzyme treatment. Baseline expression analysis by quantitative RT-PCR revealed that *BCL-2* mRNA is significantly overexpressed in MCF-7 (human breast adenocarcinoma) and HepG2 (human hepatocellular carcinoma) cells relative to HFL-1 (normal human lung fibroblasts), indicating these cell lines are suitable models for investigating *BCL-2*-targeted gene knockdown (Figure 2A).

Transfection of MCF-7 and HepG2 cells with DNZ-15 and DNZ-35a at a concentration of 200 nM resulted in a robust and statistically significant reduction in *BCL-2* mRNA levels compared to mock-transfected controls (Figure 2B). The degree of downregulation observed across both cancer lines underscores the catalytic efficiency and cross-cell-type applicability of the DNAzymes.

To further explore the downstream effects of *BCL-2* silencing, we assessed the expression of key pro-apoptotic markers regulated by mitochondrial apoptotic signaling. PUMA (p53-upregulated modulator of apoptosis) is a critical pro-apoptotic BH3-only protein that directly antagonizes anti-apoptotic *BCL-2* family members and activates BAX/BAK-dependent mitochondrial apoptosis. BFL-1 (*BCL-2*-related protein A1) is an anti-apoptotic *BCL-2* family protein that suppresses apoptosis by sequestering pro-apoptotic factors. Its transient upregulation can occur as a compensatory survival response to apoptotic stress before eventual cell death, such as in our case of *BCL-2* suppression [31,32,33,34]. Notably, in HepG2 cells, DNZ-15 treatment induced upregulation of PUMA and BFL-1 (Figure 2C). These findings suggest that the decrease in *BCL-2* expression translates into functional activation of apoptosis-related pathways.

In addition to human cancer cells, we extended our analysis to the murine 4T1 mammary carcinoma cell line, which was used in later in vivo studies. Upon transfection, both DNZ-15 and DNZ-35a suppressed *BCL-2* mRNA expression in 4T1 cells (Figure 2D). Together, these results demonstrate the consistent and effective knockdown of *BCL-2* mRNA by DNZ-15 and DNZ-35a across multiple biologically relevant cancer cell lines, including human and murine models.

### 3.3. Both the Catalytic Domain and the Substrate Recognition Regions Are Needed for DNAzyme DNZ-15 and DNZ-35a Function

**Figure 3:**
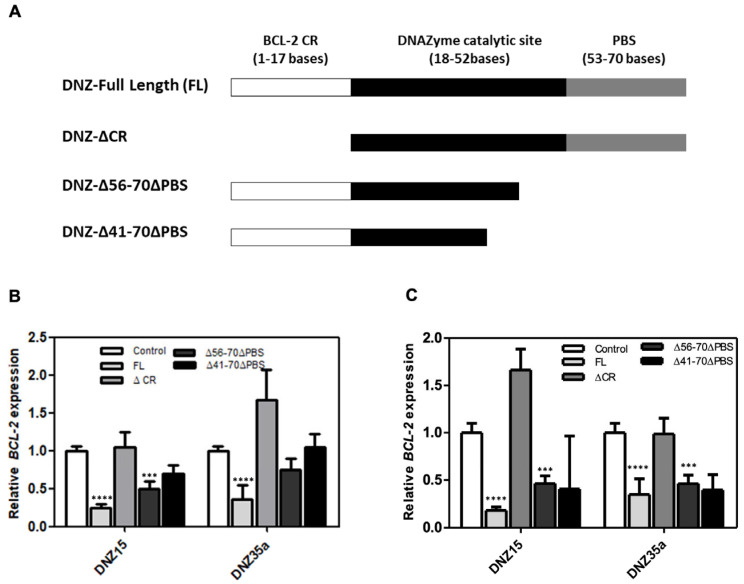
(**A**) Schematic diagram of truncated DNAzymes that were commercially synthesized. The bases deleted from the full-length (FL) DNAzyme to create various truncated products are indicated: ΔCR signifies the deletion of 17 bases from the *BCL-2* mRNA-complementary region; DNZ-Δ56–70ΔPBS indicates the deletion of the primer binding regions of the DNAzymes; and DNZ-Δ41–70ΔPBS represents the partial deletion of the catalytic part of DNZ-15 and DNZ-35a. (**B**) *BCL-2* mRNA expression measured in HepG2 (**B**) and MCF-7 (**C**) cells after transfection with various truncated and full-length DNZ15 and DNZ35a. mRNA levels are expressed relative to the control (mock-transfected). Error bars represent the mean ± SD from three separate experiments performed in triplicate. Significance levels are indicated as *** *p* < 0.001 significant and **** *p* < 0.0001 highly significant.

To better understand the structural basis for catalytic activity, we performed a detailed structure-function analysis of our lead DNAzymes, DNZ-15 and DNZ-35a. Specifically, we evaluated whether truncations in either the mRNA-binding arms or the catalytic core would impact the ability of the DNAzymes to downregulate *BCL-2* mRNA. Three constructs were created: (1) DNZ-ΔCR, in which 17 nucleotides from the *BCL-2*-complementary region were removed, effectively eliminating binding specificity; (2) DNZ-Δ56–70ΔPBS, which lacked the 3′ primer binding site and part of the structural scaffold supporting the catalytic domain; and (3) DNZ-Δ41–70ΔPBS, in which a significant portion of the core catalytic domain was deleted. The functional activity of these truncated constructs was assessed in both HepG2 and MCF-7 cells by measuring *BCL-2* mRNA expression levels post-transfection (Figure 3B,C). As expected, the full-length (FL) DNZ-15 and DNZ-35a DNAzymes significantly reduced *BCL-2* mRNA expression, validating their catalytic potency in cell-based assays.

In contrast, the DNZ-ΔCR variant, which lacked the *BCL-2*-complementary arm, showed no measurable downregulation of *BCL-2* mRNA, confirming that target recognition through Watson–Crick base pairing is essential for function. The DNZ-Δ56–70ΔPBS construct, which retained the catalytic domain but lacked essential scaffold and primer regions, showed a moderate (~0.5-fold) reduction in *BCL-2* mRNA, suggesting impaired structural integrity and reduced folding efficiency.

Most notably, the DNZ-Δ41–70ΔPBS variant—missing a significant portion of the catalytic core—exhibited only partial mRNA suppression, further reinforcing that both the catalytic domain and substrate recognition regions are indispensable for optimal activity.

These results provide strong evidence that our evolved DNAzymes function not as antisense oligonucleotides but as true nucleic acid enzymes, whose activity relies on precise secondary structure formation and proper alignment of catalytic residues. The observed differences in efficacy among the truncated variants highlight the importance of maintaining the full-length structural architecture to ensure correct folding and maximal RNA cleavage activity.

### 3.4. DNZ-15 and DNZ-35a Induce Apoptosis and Cell Death in Cancer Cells In Vitro

**Figure 4:**
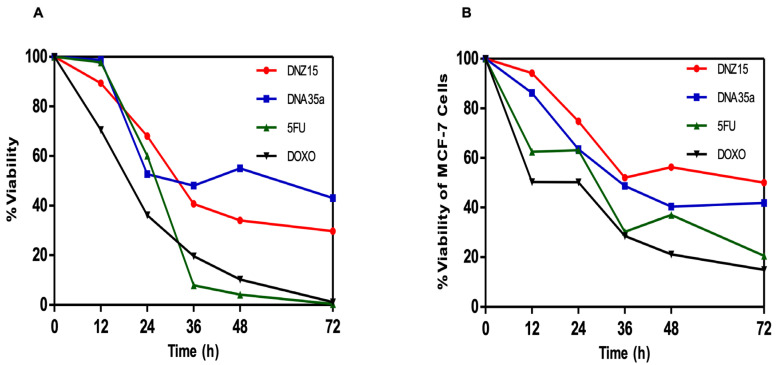
Reduction in cell viability of (**A**) HepG2 and (**B**) MCF-7 cells after treatment with 200 nM of DNZ15 and DNZ35a, along with 5-Fluorouracil (100 μM) and Doxorubicin (20 μM), as estimated using the MTT cell viability assay. The assay was repeated three times.

To determine whether the downregulation of *BCL-2* by DNZ-15 and DNZ-35a translated into functional anti-cancer effects, we assessed cell viability and apoptosis induction in vitro. MTT cell viability assays were conducted in HepG2 (human hepatocellular carcinoma) and MCF-7 (human breast cancer) cells treated with 200 nM of either DNAzyme. These experiments were repeated independently three times. The results revealed a significant, time-dependent reduction in cell viability in both cell lines over 72 h of treatment (Figure 4A,B).

In HepG2 cells, DNZ-15 induced the most pronounced cytotoxic response, with cell death of approximately 60% at 72 h, while DNZ-35a caused about 50% cell death over the same time frame. In MCF-7 cells, DNZ-15 and DNZ-35a induced 50% and 60% cell death, respectively.

To further validate that the loss of viability was due to apoptotic cell death, we performed Annexin V-FITC and Propidium Iodide (PI) staining in HepG2 cells, followed by imaging via confocal microscopy (Supplementary Figure S1). Annexin V staining identified cells in early apoptosis (green fluorescence), while PI marked late apoptotic or necrotic cells (red fluorescence). In the merged images, DNAzyme-treated cells exhibited strong green and red staining patterns, confirming apoptosis induction as the primary mode of cell death. Control cells (mock-transfected) showed minimal staining, indicating low background apoptosis.

These results clearly demonstrate that DNZ-15 and DNZ-35a possess potent in vitro anti-cancer activity by actively inducing apoptosis in *BCL-2*-overexpressing tumor cells. The ability to trigger programmed cell death through *BCL-2* silencing suggests that these DNAzymes function through a mechanism consistent with the intrinsic apoptotic pathway, restoring sensitivity to apoptotic stimuli by removing the anti-apoptotic blockade.

Importantly, this apoptotic response was consistent across two different cancer cell types—liver and breast cancer—further supporting the broad therapeutic applicability of our evolved DNAzymes. Taken together with the mRNA downregulation data, these results confirm that DNZ-15 and DNZ-35a are not only catalytically active in vitro but also biologically functional as therapeutic gene silencers, capable of suppressing tumor cell growth through apoptosis induction.

### 3.5. DNZ-15 and DNZ-35a Exhibit In Vivo Antitumor Efficacy

To evaluate the therapeutic potential of our evolved DNAzymes in a physiologically relevant setting, we tested DNZ-15 and DNZ-35a in a syngeneic orthotopic mouse model of breast cancer using 4T1 cells. Four groups were used in this experiment to assess the effects of the different treatments. The groups included the DNZ-15 treatment group, DNZ-35A treatment group, 5-Fluorouracil (5FU) treatment group, and control group (received no treatment). The control group served as a baseline to evaluate the efficacy and safety of the test compounds (DNZ-15 and DNZ-35A) in comparison to a standard chemotherapeutic agent (5FU). A single animal experimental unit was designed to assess the antitumor potential of the evolved DNAzymes.

The 4T1 model is widely recognized for its aggressive tumor growth and metastatic behavior in immunocompetent BALB/c mice, making it a clinically relevant system for assessing both efficacy and immunological tolerance of new cancer therapeutics [35,36].

**Figure 5:**
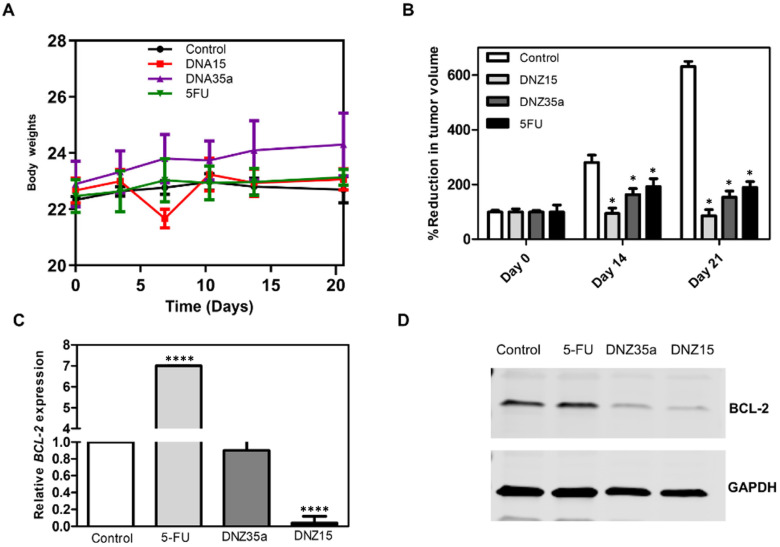
(**A**) Body weight of animals during the study. The weight of the animals in the control and drug control groups remained unchanged throughout the study period. The DNZ-35a-treated group showed an overall increase in body weight, but no such increase was observed in the DNZ-15-treated group. (**B**) Reduction in tumor volume following DNAzyme and 5-Fluorouracil treatment (Day 0 being the day of dosing, with observations on Day 10 and Day 21 post-dosing). At the end of the 21-day study period, the mean tumor volume of the control group was 600 cm^3^. The DNZ-15-treated group had a mean tumor volume of less than 100 cm^3^, and the DNZ-35a-treated group had a volume below 200 cm^3^, while the drug control-treated group had a volume near or equal to 200 cm^3^. Both DNAzymes and the 5FU drug control significantly inhibited tumor growth, with significant inhibition observed 21 days post-treatment compared to control (* p < 0.005). Data are presented as the mean ± SEM and analyzed by the paired Student’s *t*-test. (**C**) Relative *BCL-2* mRNA levels in excised tumor tissues 21 days post-treatment determined by Real-Time qPCR. *BCL-2* mRNA expression was significantly reduced in the DNZ-15 and DNZ-35a groups compared to the control and drug control groups, with the mRNA levels expressed relative to the control (**** *p* < 0.0001). (**D**) Western blot images of *BCL-2* protein expression in excised tumor tissues 21 days post-treatment. GAPDH was used to normalize *BCL-2* protein expression as a loading control.

Ten days after tumor induction, when the tumors reached a measurable and consistent volume, the animals were divided into four groups and treated with DNZ-15, DNZ-35a, 5-Fluorouracil (5-FU), or vehicle control. The DNAzymes were administered locally at the tumor site on days 10 and 15 post-implantation. The tumor volume was monitored over 21 days to evaluate the treatment effects (Figure 5B).

Both DNAzymes significantly suppressed tumor growth compared to the control group, with DNZ-15 demonstrating the most potent antitumor activity. By day 21, the average tumor volume in the DNZ-15-treated mice were reduced to below 100 mm^3^, compared to 600 mm^3^ in the untreated controls. DNZ-35a also achieved substantial tumor suppression, maintaining a tumor volume under 200 mm^3^, a result comparable to that of 5-FU-treated animals. These findings confirm that both DNAzymes are functionally active in vivo, and their antitumor efficacy is comparable to an established chemotherapeutic agent, highlighting their translational potential.

Importantly, throughout the treatment period, the body weight of the animals remained stable across all the groups, indicating minimal systemic toxicity (Figure 5A). Notably, the DNZ-35a group even showed a modest increase in body weight, further supporting the favorable safety profile of the DNAzyme treatment compared to conventional chemotherapy, which often results in weight loss due to toxicity.

To investigate whether tumor suppression was mediated by *BCL-2* silencing, we performed a molecular analysis on excised tumor tissues collected 21 days post-treatment. Quantitative real-time PCR revealed a significant reduction in *BCL-2* mRNA levels in DNZ-15-treated tumors compared to the control and 5-FU groups (*p* < 0.0001) (Figure 5C). These results strongly suggest that the DNAzymes were able to access the tumor environment, enter cells, and effectively cleave the target *BCL-2* mRNA in vivo.

Western blot analysis corroborated the transcriptional findings, demonstrating marked decreases in *BCL-2* protein expression in tumors treated with DNZ-15 and DNZ-35a (Figure 5D).

Additionally, the immunohistochemical staining of the tumor sections provided visual confirmation of reduced *BCL-2* protein levels in the DNZ-treated groups (Supplementary Figure S3). Fewer *BCL-2*-positive cells were observed in these tumors compared to the control or 5-FU-treated tissues, aligning with the Western blot data and reinforcing the conclusion that the antitumor effects of DNZ-15 and DNZ-35a were mechanistically linked to *BCL-2* knockdown.

Together, these data provide strong in vivo evidence that DNZ-15 and DNZ-35a induce tumor regression by specifically silencing *BCL-2* expression at both the mRNA and protein levels. The magnitude of tumor inhibition, combined with the favorable safety profile and mechanism-specific activity, positions these DNAzymes as promising candidates for further development as targeted nucleic acid therapeutics for cancer treatment.

## 4. Discussion

Our study presents a novel and effective strategy for the in vitro evolution and characterization of trans-acting RNA-cleaving DNAzymes specifically targeting the *BCL-2* mRNA—a key anti-apoptotic gene that is overexpressed in many human cancers. Unlike traditional in vitro selection protocols that rely on covalent attachment of the RNA substrate to the DNAzyme library, we employed a more physiologically relevant trans-acting selection approach using a native, biotinylated 50-nucleotide fragment of *BCL-2* mRNA. This design allowed us to select DNAzymes that bind and cleave their targets through natural base-pairing interactions, reflecting the intracellular environment more closely.

From this selection, two DNAzymes, DNZ-15 and DNZ-35a, emerged as potent candidates, displaying robust cleavage activity in vitro and significant *BCL-2* knockdown in liver carcinoma and breast cancer cells. The DNAzyme-mediated suppression of *BCL-2* expression led to the upregulation of pro-apoptotic markers PUMA and BFL-1 and a reduction in cell viability—demonstrating a direct functional consequence of gene silencing. Importantly, loss-of-function experiments using truncated variants confirmed that both the mRNA-complementary region and catalytic core are essential for DNAzyme activity, emphasizing the precision of these evolved molecules.

These results build upon a foundational body of work on RNA-cleaving DNAzymes, initially pioneered by Breaker and Joyce in 1994 [12] and later refined by Santoro and Joyce with the introduction of the well-known 10–23 and 8–17 motifs [37]. The therapeutic utility of DNAzymes has been explored in various disease contexts, including targeting VEGF in age-related macular degeneration [38], c-Jun in skin cancer [39], and *BCL-2* in hematologic malignancies [40]. However, despite encouraging preclinical data, few DNAzymes have progressed into advanced clinical development, often limited by delivery challenges and suboptimal in vivo stability.

Compared to prior studies targeting *BCL-2*, such as the 10–23 DNAzyme explored by Yang et al. [41], which showed moderate mRNA suppression in leukemia models, our evolved DNZ-15 and DNZ-35a DNAzymes demonstrate improved catalytic efficiency (35% and 30% cleavage, respectively) under near-physiological conditions. Moreover, we extended the validation beyond cell lines to a syngeneic mouse model using 4T1 mammary carcinoma cells—an aggressive, immunocompetent model that more accurately reflects tumor–immune interactions. In vivo administration of DNZ-15 and DNZ-35a significantly suppressed tumor growth, with effects comparable to the chemotherapeutic agent 5-Fluorouracil (5-FU), but potentially with fewer systemic side effects, as evidenced by a stable body weight and the absence of gross toxicity.

The molecular analysis of excised tumors confirmed the silencing of *BCL-2* at both the mRNA and protein levels. These results highlight the potential of DNAzymes as viable alternatives to RNAi, antisense oligonucleotides (ASOs), or small-molecule inhibitors [42,43]. While siRNAs and ASOs can efficiently degrade target mRNAs via RNase H or RISC pathways, they often suffer from off-target effects, innate immune activation, and delivery barriers in vivo [44,45]. In contrast, DNAzymes—particularly those evolved for trans-acting activity—offer higher stability in serum, reduced immunogenicity, and a clear catalytic mechanism of action.

Importantly, our in vitro selection platform is modular and scalable. By simply modifying the complementary binding arms, it can be adapted to target virtually any mRNA sequence of interest. This provides a flexible tool not only for therapeutic development but also for functional genomics and gene validation in diverse biological systems. Of course, as the field is moving forward, regulatory approval of DNAzyme-based therapeutics will require careful consideration of their safety, specificity, and delivery mechanisms. Unlike traditional small molecules, DNAzymes are classified as nucleic acid-based drugs and thus fall under regulatory frameworks similar to antisense oligonucleotides and gene therapies. Key considerations include demonstrating sequence-specific cleavage with minimal off-target effects, validating biodistribution and pharmacokinetics, and ensuring consistent manufacturing standards for clinical-grade oligonucleotides. Additionally, regulatory agencies will expect comprehensive preclinical toxicology studies and immunogenicity assessments before the initiation of human trials. While further studies are warranted to address these issues and optimize delivery—potentially using lipid nanoparticles, aptamer conjugation, or viral vectors—the current findings provide a compelling proof of concept for DNAzyme-based cancer therapeutics, particularly for genes like *BCL-2*, where dysregulation plays a central role in tumor survival and chemoresistance [46].

In the future, this approach may be extended to other members of the *BCL-2* family, such as MCL-1 or BCL-XL, or to non-apoptotic gene targets in areas like viral replication, neurodegeneration, and inflammatory diseases. The combination of trans-acting DNAzyme design with evolving delivery technologies could mark a turning point in the clinical translation of DNA-based therapeutics.

## 5. Conclusions

We report the successful evolution of trans-acting RNA-cleaving DNAzymes using a novel in vitro selection approach that employs natural mRNA sequences as substrates. The selected DNAzymes, DNZ-15 and DNZ-35a, effectively downregulate *BCL-2* expression in human and mouse cancer models, induce apoptosis, and suppress tumor growth in vivo. Unlike conventional methods, our strategy does not require covalent substrate tethering, enabling the discovery of DNAzymes that act under more physiologically relevant conditions. This work not only demonstrates the therapeutic potential of DNAzymes targeting anti-apoptotic pathways but also establishes a scalable, generalizable framework for evolving gene specific DNAzymes for research and clinical applications. Future studies will focus on optimizing the delivery of DNAzymes in vivo and expanding this approach to other oncogenic targets. Additionally, preclinical validation in animal models will be critical to assess pharmacokinetics, biodistribution, and long-term safety of the DNAzymes before advancing to human trials. If successful, this approach could offer a novel strategy to overcome chemotherapy resistance in *BCL-2*-dependent tumors, addressing a significant unmet need in oncology.

## Data Availability

The datasets used in this analysis are available from the corresponding authors upon reasonable request.

## Supplementary Materials

The following supporting information can be downloaded at: https://www.mdpi.com/article/10.3390/cells14130945/s1, Table S1: List of primers used for the RT-PCR analysis; Table S2: DNAzymes sequences; Figure S1: Apoptotic cell imaging using confocal microscopy. HepG2 cells (4 X 104) were seeded on poly-L-lysine-coated chamber slides one day before transfection. 24 h after DNAzyme transfection (200nM), cells were washed with cold 1X PBS followed by the addition of 5 μL of FITC-Annexin V solution (Molecular Probes) and 100 μg/mL of Propidium Iodide solution (Molecular Probes) to each chamber. Cells were incubated for 15 min at room temperature after which cells were washed once with 1X Annexin binding buffer (Molecular Probes). Apoptotic/dead and live cells were imaged by Leica Microsystems confocal microscope (TCS SP8) [24,25].; Figure S2: Animal tumour images during the study period. Syngeneic 4T1 model. Animal tumour volumes in mm3. A decreased tumour growth was observed in the DNZ-15 treated group than in the DNZ-35a and drug control and control groups. This procedure was performed once for each animal, at the beginning of the experiment, to initiate tumor formation. The 4T1 cell line is a well-established, aggressive murine breast cancer model that closely mimics human triple-negative breast cancer in terms of tumor growth and metastatic potential. Injecting cells into the mammary fat pad allows for orthotopic tumor development, providing a physiologically relevant tumor microenvironment. The removal of fur ensures accurate cell delivery and reduces the risk of contamination or injection errors.; Figure S3: Tumor tissues were sliced at a diameter of 5 μm with histotome. Tissue sections were fixed in 10% formalin and embedded in paraffin wax. Sections were incubated with Anti- *BCL-2* primary antibody (Merck) overnight at 40C followed by incubation with HRP conjugated secondary antibody (Merck) for 2h at room temperature. *BCL-2* expression was detected by adding a TMB substrate and slides were imaged in the microscope (Leica Microsystems, Mannheim, Germany).
