# Supplementary material for: Novel In Vitro Selection of Trans-Acting BCL-2 mRNA-Cleaving Deoxyribozymes for Cancer Therapy
Source: Cells. 2025 Jun 20;14(13):945. doi: 10.3390/cells14130945 (PMC12248697; doi:10.3390/cells14130945)
Supplement: Supplementary file 1 [file cells-14-00945-s001.zip › cells-3674290-supplementary.pdf]

Supplementary Data

Table S1 - list of primers used for the RT-PCR analysis.

Target Name	Species	Primer Sequence	T_m
BCL-2	*Homo sapiens*	Fwd: 5’- ATGTGTGTGGAGAGCGTCAA-3’<br>Rev: 5’- ACAGTTCCACAAGGCATCC-3’	56.6^0C<br>55.6^0C
BCL-2	*Mus musculus*	Fwd: 5’-CTGGCATCTTCTCCTTCCAG-3’<br>Rev: 5’-GACGGTAGCGACGAGAGAAG-3’	55.1^0C<br>57^0C
18s	*Homo sapiens*	Fwd: 5’- CGGCGACGACCCATTCGAAC-3’<br>Rev: 5’- GAATCGAACCCTGATTCCCCGTC-3’	61.1^0C<br>59.6^0C
18s	*Mus musculus*	Fwd: 5’-GCAATTATTCCCCATGAACG-3’<br>Rev: 5’-GGCCTCACTAAACCATCCAA-3’	51.9^0C<br>54.8^0C
PUMA	*Homo sapiens*	Fwd: 5’- GGAGCAGCACCTGGAGTG-3’<br>Rev: 5’- TACTGTGCGTTGAGGTCGTC-3’	58.2^0 C<br>57.0^0 C
BFL-1	*Homo sapiens*	Fwd: 5’- TTACAGGCTGGCTCAGGACT-3’<br>Rev: 5’- CCCAGTTAATGATGCCGTCT-3’	58.2^0 C<br>54.8^0 C

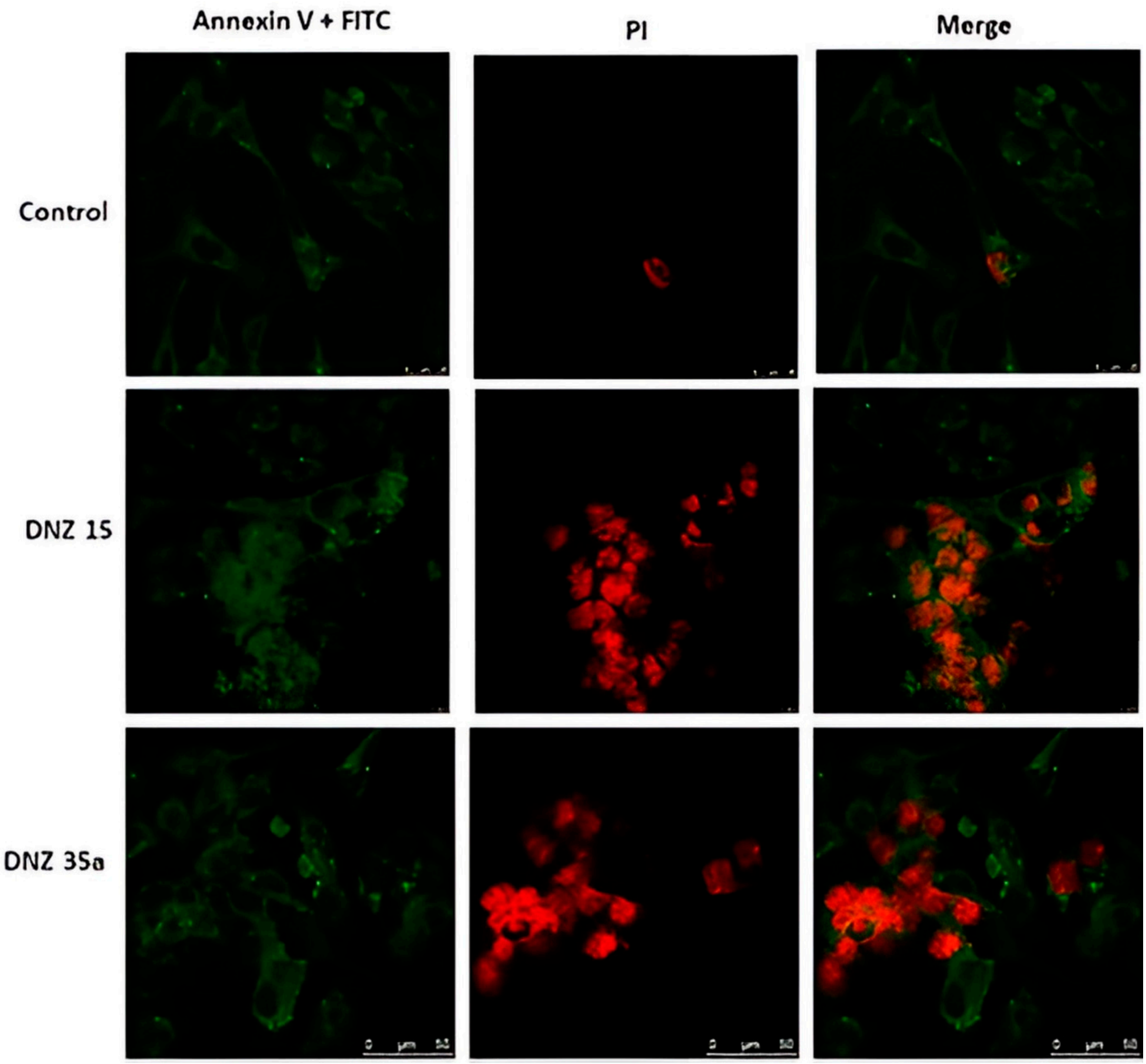

Supplementary Figure S1.

Apoptotic cell imaging using confocal microscopy.

HepG2 cells (4 X 10^4) were seeded on poly-L-lysine-coated chamber slides one day before transfection. 24 h after DNAzyme transfection (200nM), cells were washed with cold 1X PBS followed by the addition of 5µl of FITC-Annexin V solution (Molecular Probes) and 100µg/ml of Propidium Iodide solution (Molecular Probes) to each chamber. Cells were incubated for 15 min at room temperature after which cells were washed once with 1X Annexin binding buffer (Molecular Probes). Apoptotic/dead and live cells were imaged by Leica Microsystems confocal microscope (TCS SP8) [24-25].

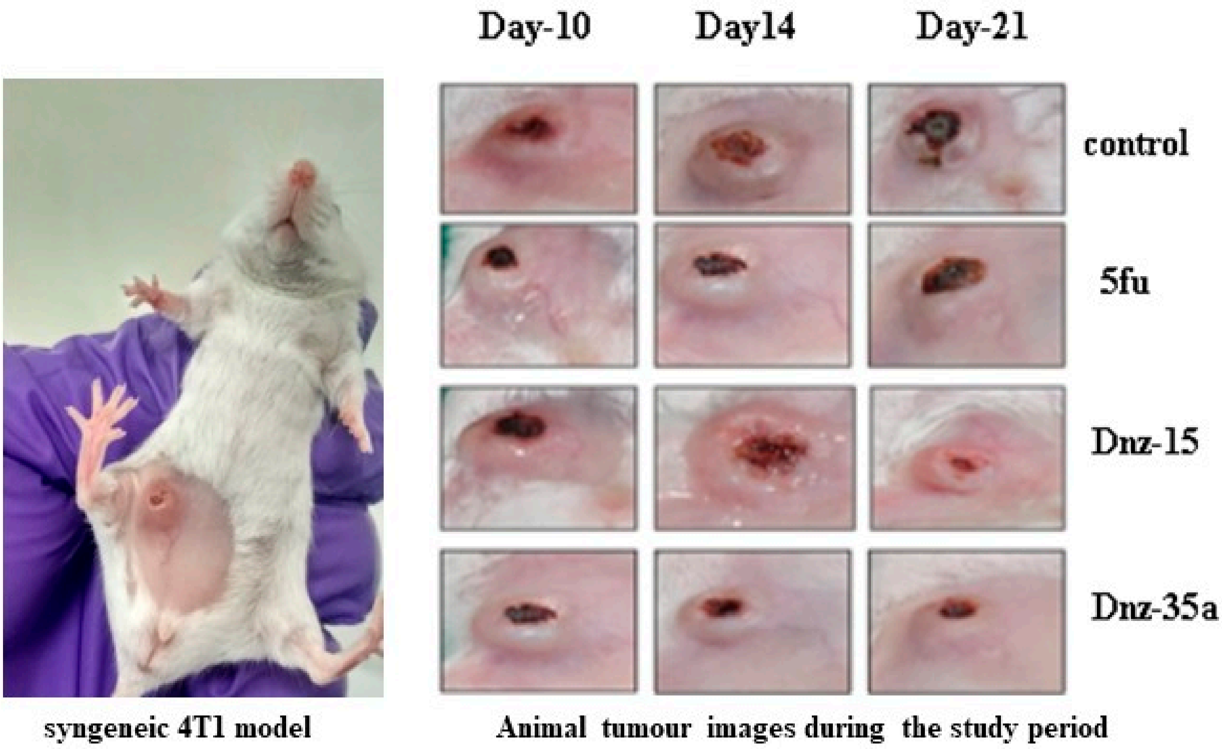

Supplementary Figure S2 Animal tumour images during the study period. Syngeneic 4T1 model. Animal tumour volumes in mm^{3}. A decreased tumour growth was observed in the DNZ-15 treated group than in the DNZ-35a and drug control and control groups. This procedure was performed once for each animal, at the beginning of the experiment, to initiate tumor formation. The 4T1 cell line is a well-established, aggressive murine breast cancer model that closely mimics human triple-negative breast cancer in terms of tumor growth and metastatic potential. Injecting cells into the mammary fat pad allows for orthotopic tumor development, providing a physiologically relevant tumor microenvironment. The removal of fur ensures accurate cell delivery and reduces the risk of contamination or injection errors.

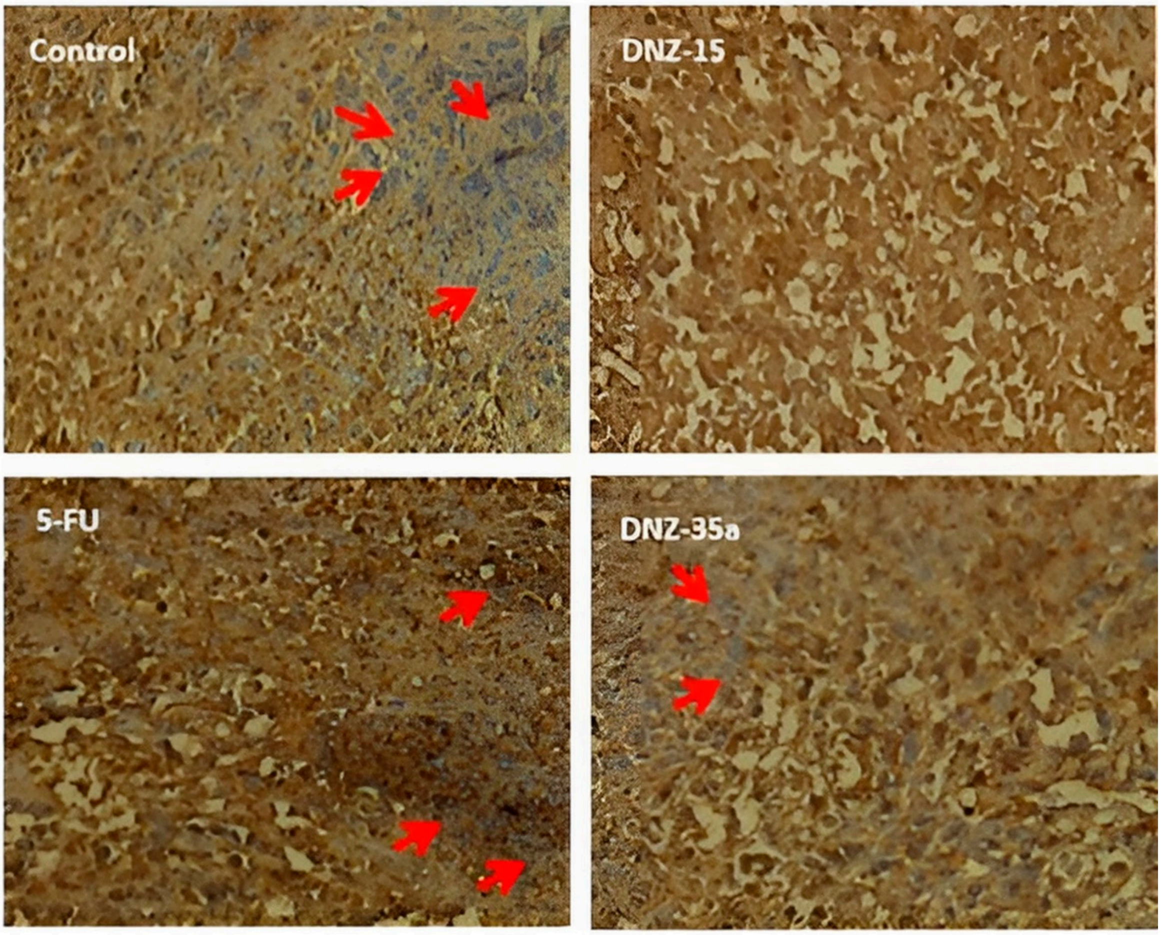

Supplementary Figure S3.

Tumor tissues were sliced at a diameter of 5µm with histotome. Tissue sections were fixed in 10% formalin and embedded in paraffin wax. Sections were incubated with Anti- *BCL-2* primary antibody (Merck) overnight at 4^0C followed by incubation with HRP conjugated secondary antibody (Merck) for 2h at room temperature. *BCL-2* expression was detected by adding a TMB substrate and slides were imaged in the microscope (Leica Microsystems, Mannheim, Germany).

Table S2 DNAzymes sequences.

S.no	Sequence ID	DNAzyme Sequence	Tm
1.	DNZ 15	5’GGCTGGATGGGGCGTGTACCACCACACTGTCCGACCCACCCC CAGACACACCCCAACGGTGCGGACAGCG 3’	77.3^0 C
2.	DNZ 22	5’GGCTGGATGGGGCGTGTGGACCACAGACATCACCCCGCAGCC CCAGCCCTGGGCAACGGTGCGGACAG 3’	78.6^0 C
3.	DNZ 24	5’GGCTGGATGGGGGCGTGTCCATGCAGCAACCAACCACAGGCA AACCACGCGTCCCAACGGTGCGGACAGCG 3’	77.0^0 C
4.	DNZ 35a	5’GGCTGGATGGGGCGTGTCGACCCAGACCCACCACACGCACAC CTCCCGCTGCCCAACGGTGCGGACAGCG 3’	78.3^0 C
5.	DNZ 35b	5’GGCTGGATGGGGCGTGTAGCGCATCGGAGCATGCGTCGTCCA AGGTGTGTGGGCAACGGTGCGGAC 3’	77.0^0 C

Animal studies

Objectives.

This study aimed to evaluate whether evolved DNAzymes could significantly inhibit tumor growth in "BALB/c mice with xenografted tumors". The primary objective was to determine if DNAzyme treatment reduces tumor volume compared to controls, with secondary assessments of survival and molecular markers expression levels of the BCL-2, PUMA, NOXA mRNA levels by RT-PCR. We specifically tested the hypothesis that DNAzyme treatment would achieve a reduction in tumor volume based on preliminary data. The experimental design focused on quantifying treatment efficacy through longitudinal tumor measurements. Findings from this preclinical study provide critical evidence for further therapeutic development of DNAzymes against solid tumors.

Animal Housing and husbandry conditions.

The animals were housed in an SPF (specific pathogen-free) facility under strictly controlled environmental conditions, maintained at 22±2°C temperature, 55±10% humidity, with a 12-hour light/dark cycle (7 AM-7 PM) and 10-15 air changes per hour. They were kept in polycarbonate cages with corn cob bedding, changed twice weekly. Daily health checks were performed by trained staff, and tumor measurements were conducted throughout the study period. All procedures were approved by our Institutional Animal Care and Use Committee 1996/PO/Re/S/17/CPCSEA.

Animal care and monitoring.

Isoflurane anaesthesia was used during the sacrifice procedure to minimize pain and distress. All procedures were conducted in accordance with ethical guidelines for animal welfare. During the study, we observed some expected adverse events related to tumor growth in the animal model. Specifically, in cases where tumors developed to larger sizes (exceedingly approximately 10% of body weight), some animals experienced physical friction between the tumors and cage surfaces during normal movement. This occasionally resulted in superficial abrasions or mild ulceration at the tumor site. To mitigate these effects, we implemented additional measures like more frequent cage monitoring (twice daily for affected animals. For this study, predefined humane endpoints were established to ensure animal welfare and minimize suffering. Animals were monitored twice daily (morning and evening) for signs of distress, tumour condition and animal movement in the cages.

Interpretation/ scientific implications

The study confirmed that DNAzymes effectively reduced tumor growth supporting our hypothesis. While results align with similar therapies in literature, some animals developed tumor-related complications at larger sizes, suggesting room for improvement in treatment protocols. These findings demonstrate promising anti-cancer potential but highlight the need for further optimization before clinical use. In our study the small sample size may limit statistical power. Tumor friction in some animals introduced potential bias. The xenograft model may not fully replicate human tumor biology. Measurement variability could affect precision.

Generalisability.

While this BALB/c animal study shows promising DNAzyme efficacy, human translation may be limited by species differences in tumor biology. Results could vary with alternative dosing or delivery methods. Further testing in advanced models is needed to assess clinical relevance. A study protocol was developed before experimentation but was not formally registered. The full methodology is reported transparently in this manuscript to enable reproducibility. The datasets generated and analysed during this study, including raw tumor measurements, treatment protocols, and statistical analyses, are not publicly available due to ethical restrictions involving animal research approvals and institutional policies.
